# Best foot forward: podiatrists’ insight and awareness of melanoma of the foot—a questionnaire study

**DOI:** 10.1093/skinhd/vzaf008

**Published:** 2025-04-16

**Authors:** Brian Nolan, Cathal O’Connor, Michelle Murphy

**Affiliations:** Dermatology, South Infirmary Victoria University Hospital, Cork, Ireland; Dermatology, South Infirmary Victoria University Hospital, Cork, Ireland; Dermatology, University College Cork, Cork, Ireland; INFANT Research Centre, University College Cork, Cork, Ireland; Dermatology, South Infirmary Victoria University Hospital, Cork, Ireland; Dermatology, University College Cork, Cork, Ireland

## Abstract

A significant proportion of melanomas arise on the lower limb, which may present to podiatrists. In this study, two-thirds (67.6%) of podiatrists reported reviewing pigmented lesions, and two-thirds (69.6%) reported identifying a lesion suspicious for melanoma. Only 57.8% of podiatrists felt comfortable contacting their patient’s general practitioner, and most podiatrists (82.2%) felt they received inadequate training in melanoma.

Dear Editor, Melanoma is the fourth most common cancer in the Republic of Ireland, representing 4% of cancers in male patients and 5% in female patients.^1^ The incidence of melanoma is increasing worldwide, credited to earlier detection of thin melanomas.^2^ Nearly 30% of melanomas affect the lower limbs,^3–5^ and melanoma of the foot or toenails can masquerade as ulcers, congealed blood and inflammatory conditions,^6,7^ potentially delaying diagnosis and increasing mortality. Allied healthcare professionals (HCPs) can play a key role in melanoma detection.^8^ We aimed to assess the experience and confidence of podiatrists in diagnosing melanoma of the lower limb.

A 17-question web survey was designed by the authors to assess the demographics, experience and confidence of podiatrists in relation to melanoma of the lower limb. The survey investigated the frequency of presentation of suspicious lesions, the perceived characteristics of suspicious lesions, the current management of suspicious lesions, and suggestions to improved detection of melanoma among podiatrists. The survey was distributed to all members of the Irish Chiropodists/Podiatrists Organisation (ICPO) (Supplementary File S1). The survey was conducted using Google Forms and data were exported to SPSS for analysis.

The survey had a 61.8% response rate (*n* = 247), with 80.2% (*n* = 198) having at least 20 years’ experience, with an average of 30 patients seen per week. Two-thirds (67.6%, *n* = 167) reported being asked to review pigmented lesions, and 41.3% (*n* = 102) reported frequently examining pigmented lesions. Over two-thirds (69.6%, *n* = 172) reported identifying a lesion suspicious for melanoma. Of 66 podiatrists who reported the most frequent site where they identified suspicious pigmented lesions, 20% (*n* = 13) noted the plantar foot to be the most common site, followed by toes and toenails (18%, *n* = 12 each). Of 105 podiatrists who selected the key feature that they assessed when examining suspicious pigmented lesions, 24.8% (*n* = 26) assessed pigmentation, 14.3% (*n* = 15) assessed size, and 11.4% (*n* = 12 each) assessed evolution, appearance, borders and shape (Figure 1). Of 50 podiatrists who described their routine management of suspicious lesions, 88% (*n* = 44) advised general practitioner (GP) referral, 8% (*n* = 4) advised dermatology referral, 2% (*n* = 1) took a biopsy and 2% (*n* = 1) took photographs for ‘mole-mapping’. Of 172 podiatrists who reported identifying lesions concerning for melanoma, just 17.4% (*n* = 30) reported receiving feedback on outcomes from subsequent medical assessment. Only 57.9% (*n* = 143) of all podiatrists reported feeling comfort­able contacting a patient’s GP if they identified a suspicious lesion, only 54.7% (*n* = 135) reported feeling comfortable discussing photoprotection with patients and only 44.5% (*n* = 110) felt comfortable discussing tanning or sunbeds. Most podiatrists (82.2%, *n* = 203) felt they received inadequate training in melanoma during their training. Suggested methods for improving their management of melanoma included undergraduate teaching (12.6%, *n* = 31), improved referral pathways (3.2%, *n* = 8), educational seminars (1.6%, *n* = 4) and direct feedback from GPs/dermatologists regarding outcomes (1.6%, *n* = 4).

**Figure 1:**
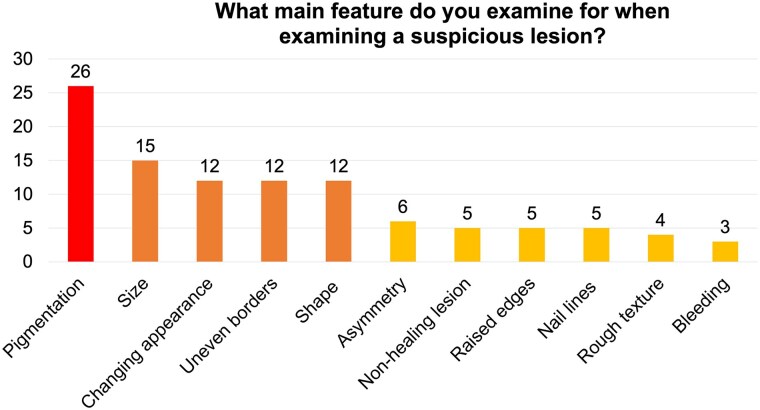
Features that podiatrists (*n* = 105) reported examining for when reviewing suspicious/concerning lesions on the foot.

This study shows that most podiatrists have been asked to review pigmented lesions, and that most have identified a lesion that they believed to represent a melanoma. Podiatrists may be the first healthcare providers to review melanoma of the lower limb, given their role as community HCPs, and given pressure on general practice and hospital services. Podiatrists identified a concerning lack of confidence and training in dealing with melanoma of the lower limb. Most podiatrists said that they would advise their patient to attend their GP, and a lack of a direct pathway from podiatrists to GPs or specialty care was highlighted. Moreover, nearly half of podiatrists did not feel comfortable directly contacting a GP to refer a patient. Even fewer had received an update on a patient with a suspicious pigmented lesion after advising review with their GP, a missed opportunity for continuous professional development. In the UK, NICE (National Institute for Health and Care Excellence) guidelines emphasize that lesions suspicious for melanoma should be seen in specialized pigmented lesion clinics within 2 weeks of detection. The referral to a specialist melanoma service may be delayed if the patient does not attend their GP in a timely manner. Many of these patients are older, and may have lower health literacy and be at greater risk of being lost to follow-up if referral mechanisms are convoluted. Health systems in other regions may facilitate direct referrals from podiatry to specialist pigmented lesion clinics.

Strengths of this study include a high response rate from this national professional society and the lack of previous work in this area. Limitations include the lack of outcome data relating to the patients seen by the respondents and the lack of deeper qualitative characterization of surprising findings, such as the discomfort felt around contacting GPs directly.

This research highlights a disconnect in communication between community-based podiatrists, GPs and melanoma services. Avenues for future research or quality improvement include strategies for optimizing management of pigmented lesions in podiatry such as educational seminars or dermoscopy training, improved referral pathways and other policies to enhance detection of melanoma of the lower limb. Podiatrists have a key role in the early identification of melanoma. Dermatologists should support podiatrists with education on assessment and management of suspicious lesions of the lower limb, facilitating timely and efficient referral.

## Supplementary Material

vzaf008_Supplementary_Data

## Data Availability

The data underlying this article will be shared on reasonable request to the corresponding author.
