# Correction to: Best foot forward: podiatrists’ insight and awareness of melanoma of the foot—a questionnaire study

**DOI:** 10.1093/skinhd/vzaf117

**Published:** 2026-01-09

This is a correction to: Brian Nolan, Cathal O’Connor, Michelle Murphy, Best foot forward: podiatrists’ insight and awareness of melanoma of the foot—a questionnaire study, *Skin Health and Disease*, Volume 5, Issue 3, June 2025, Pages 240–241, https://doi.org/10.1093/skinhd/vzaf008

The following changes have been made to the originally published manuscript.

In the second paragraph of the the the article, the third sentence has been emended to read: “The survey was distributed to all members of the Irish Chiropodists/Podiatrists Organisation (ICPO)[…]” instead of: “The survey was distributed to all 400 members of The Society of Chiropodists and Podiatrists of Ireland, the all-island professional body for podiatrists in Ireland[…]”. The authors apologise for the oversight.

