# Supplementary material for: Best foot forward: podiatrists’ insight and awareness of melanoma of the foot—a questionnaire study
Source: Skin Health Dis. 2025 Apr 16;5(3):240–1. doi: 10.1093/skinhd/vzaf008 (PMC12202858; doi:10.1093/skinhd/vzaf008)
Supplement: vzaf008_Supplementary_Data [file vzaf008_supplementary_data.pdf]

An Insight into the Awareness of Chiropodists & Podiatrists into Melanoma of the Foot

My name is Brian Nolan - I am an Senior House Officer Doctor currently working in CUH - I have an interest in Dermatology and I am interested at examining the important role Chiropodists and Podiatrists play in Melanoma care.

I would really appreciate you answering the questions below.

Note all answers are entirely voluntary and anonymous -

any questions please do not hesitate to contact me at: brianmichaelnolan@gmail.com

Thank you.

drcathaloconnor@gmail.com Switch account

Not shared

* Indicates required question

Thank you for helping me with this survey. All data is strictly anonymous. What is your job?

- ◯ Chiropodist
- ◯ Podiatrist
- ◯ Other
- ◯ Other:

How many years' experience do you have in your field as a Chiropodist/Podiatrist? (Approximately)

- 0-5 years
- 5-10 years
- 10-20 years
- 20 years+

What would you say is the average number of patients you see per week? Can write 'N/A' if prefer not to disclose.

Your answer

Do you feel comfortable discussing Sun Care such as sunscreen with your clients?

- Yes
- No
- Other:

Do you feel comfortable discussing Sun Beds/Tanning with your clients?

- Yes
- No

Do you think you received satisfactory training in Melanoma of the Foot during your training?

- Yes
- No

Have you had clients ask you to examine suspicious/concerning lesions on the foot before?

- Yes
- No
- Other:

If yes, how frequently would you say this occurs? (Approximately)

- Very Frequently
- Frequently
- Sometimes
- Infrequently
- Never

In your career, have you encountered lesions on the foot which caused you concern that they may represent melanoma/skin cancer?

- Yes
- No
- Other:

Approximately how many times (number) have you encountered lesions on the foot which concerned you for melanoma?

Your answer

What steps did you take when you identified a suspicious lesion on the foot?

Your answer

Where on the foot is the commonest spot you have noted suspicious lesions?

Your answer

If you have advised GP presentation in the past due to a suspicious lesion, did you hear back the outcome of this?

- Yes I heard back
- No I did not hear back
- I have not advised GP presentation due to a suspicious lesion before
- Other:

Do you feel comfortable asking a patient to present to his/her GP if you note a suspicious lesion on the foot?

- Yes
- No
- Maybe

What do you look for on suspicious lesions on the foot that would make you concerned for a Melanoma? *

- Size of lesion
- Changing lesion appearance
- Pigmentation varying
- Uneven borders
- Asymmetry
- Non-healing lesion
- Shape and diameter of lesion
- Bleeding
- Raised edges
- Nail lines
- Textural change
- Other:

What steps do you think could be taken to support Podiatrists/Chiropodists in identifying suspicious lesions of the foot?

Your answer

Have you any further comments/opinions as to the role Chiropodists/Podiatrists play in the detection of melanoma of the foot?

Your answer

I really appreciate your help in completing this survey. All data is strictly anonymous. Do you consent to me using these responses in a research project?

- Yes
- No

Submit

Clear form

Never submit passwords through Google Forms.

This content is neither created nor endorsed by Google. Report Abuse - Terms of Service - Privacy Policy

Google Forms
